# Membrane Vesicles for Nanoencapsulated Sulforaphane Increased Their Anti-Inflammatory Role on an In Vitro Human Macrophage Model

**DOI:** 10.3390/ijms23041940

**Published:** 2022-02-09

**Authors:** Lucía Yepes-Molina, María Isabel Pérez-Jiménez, María Martínez-Esparza, José A. Teruel, Antonio J. Ruiz-Alcaraz, Pilar García-Peñarrubia, Micaela Carvajal

**Affiliations:** 1 Aquaporins Group, Centro de Edafología y Biología Aplicada del Segura (CEBAS-CSIC), Campus de Espinardo, 30100 Murcia, Spain; lyepes@cebas.csic.es (L.Y.-M.); marisaperez-12@hotmail.com (M.I.P.-J.); 2 Departamento de Bioquímica y Biología Molecular (B) e Inmunología, Facultad de Medicina, IMIB and Regional Campus of International Excellence “Campus Mare Nostrum”, Universidad de Murcia, 30100 Murcia, Spain; ajruiz@um.es (A.J.R.-A.); pigarcia@um.es (P.G.-P.); 3 Departamento de Bioquímica y Biología Molecular A, Facultad de Veterinaria, Universidad de Murcia, Campus de Espinardo, 30100 Murcia, Spain; teruel@um.es

**Keywords:** broccoli, inflammation, macrophage, membrane vesicles, nanocarrier, sulforaphane

## Abstract

At present, there is a growing interest in finding new non-toxic anti-inflammatory drugs to treat inflammation, which is a key pathology in the development of several diseases with considerable mortality. Sulforaphane (SFN), a bioactive compound derived from *Brassica* plants, was shown to be promising due to its anti-inflammatory properties and great potential, though its actual clinical use is limited due to its poor stability and bioavailability. In this sense, the use of nanocarriers could solve stability-related problems. In the current study, sulforaphane loaded into membrane vesicles derived from broccoli plants was studied to determine the anti-inflammatory potential in a human-macrophage-like in vitro cell model under both normal and inflammatory conditions. On the one hand, the release of SFN from membrane vesicles was modeled in vitro, and two release phases were stabilized, one faster and the other slower due to the interaction between SFN and membrane proteins, such as aquaporins. Furthermore, the anti-inflammatory action of sulforaphane-loaded membrane vesicles was demonstrated, as a decrease in interleukins crucial for the development of inflammation, such as TNF-α, IL-1β and IL-6, was observed. Furthermore, these results also showed that membrane vesicles by themselves had anti-inflammatory properties, opening the possibility of new lines of research to study these vesicles, not only as carriers but also as active compounds.

## 1. Introduction

Inflammation exerts a role in the development of several diseases and disorders, which are some of the most important scientific discoveries in the field of health research. These diseases cause considerable morbidity and contribute to early mortality [1], and include cardiovascular diseases, obesity, type 2 diabetes, some cancers and cerebrovascular stroke [2]. Finding the best way to reduce the risk of inflammation-related diseases is, therefore, a major issue. The inflammatory process is a protective response of an organism to harmful stimuli, such as pathogens, damaged cells or irritants, in order to maintain homeostasis. This process can be acute when inflammation is developed within minutes or hours and finally resolved when a functional status is restored, or chronic when acute inflammation is not resolved [2,3]. Inflammation is regulated by several immune cells, mainly neutrophils and macrophages, and soluble molecules, including pro-inflammatory cytokines, such as tumor necrosis factor alpha (TNF-α), interleukin 1β (IL-1β) or IL-6 [4]. Nonsteroidal anti-inflammatory drugs (NSAIDs) or corticosteroids are frequently used to treat this pathology, regardless of their extensive and serious side effects (ulcers, hemorrhages or nephrotoxicity) when taken for a long period [5]. Hence, there is a need to find new anti-inflammatory drugs with less or preferably no toxicity. Since the pro-inflammatory cytokines listed above are important in the initiation and maintenance of inflammation [6], targeting inhibitors of these molecular pathways involved in the production of these cytokines could be a good strategy [7,8,9].

In this regard, the study of plant-derived compounds to manage chronic inflammation has increased in recent years. Since the early 1990s, numerous pieces of evidence have demonstrated the health benefits associated with the consumption of cruciferous vegetables [10,11,12]. These healthy properties referred to glucosinolates (GLS) and their derivatives, isothiocyanates (ITC), which are produced by the action of the enzyme myrosinase [13]. Sulforaphane (SFN) (*R*-1-isothiocyanato-4-methylsulfinyl butane), a hydrolysis compound of the GLS glucoraphanin (GRA), is the most studied ITC and is predominantly found in *Brassica oleracea* L. var. *Italica* (broccoli). In this sense, the anti-inflammatory action of broccoli was shown in overweight adult subjects, where, after 70 days of sprouts broccoli consumption, a decrease in anti-inflammatory markers, such as IL-6 and C-reactive protein, was found [14]. Specifically, SFN has been noted for its antioxidant, apoptosis-inducing and anti-inflammatory effects [10,15]. In addition, SFN has great potential in the prevention and treatment of cancer in which inflammation plays a key role [16]. Zhang et al. [17] described SFN as an inductor of phase II detoxification enzymes, which promote the antioxidant response and take part in the suppression of pro-inflammatory responses [18]. Specifically, SFN is an inducer of the Nrf2 transcription factor, which is responsible for the transcription of genes involved in antioxidant activities or anti-inflammatory pathways [19,20]. One of the most powerful actions of SFN is the regulation of the inflammatory response through the inhibition of the nuclear factor kappa B (NFκB) pathway [21], which is key in the transcription of pro-inflammatory cytokine genes in response to stimuli [22].

Despite the potential effects of SFN, its actual clinical use as an anti-inflammatory drug presents difficulties due to its poor stability and short half-life, as with most natural compounds [23]. However, it was revealed that nanocarriers, such as liposomes, can solve stability-related problems. Furthermore, the stability of liposomes, along with the retention of their content, has long been described as a desirable feature for successful drug delivery to diseased tissues [24]. As for SFN, the current literature reveals that its encapsulation significantly improves its stability [25], enhances its efficacy, enables its sustained release into cells [26] and improves its anticancer activity in melanoma cells [27] or its anti-inflammatory activity in acute or chronic models of rheumatoid arthritis [28].

Liposomes are organic nanocarriers, characterized by high biocompatibility and a powerful hydrophilic or hydrophobic drug-loading capacity [29,30]. An encapsulation system similar to liposomes uses proteoliposomes, which consist of proteins and lipids [31,32]. The protein of proteoliposomes provides additional stability due to the specific lipid–protein interaction, as a lipid–protein environment similar to native membranes is simulated [33,34]. In addition to carrying out in vitro syntheses of proteoliposomes [32], several studies focused on proteoliposomes from natural sources, including extracellular vesicles or membrane vesicles to use them as nanocarriers in different biotechnological applications [31,35,36,37,38,39].

Considering membrane vesicles, plants are a suitable source of this type of nanocarriers. Our previous studies revealed the advantages of membrane vesicles from plants, specifically from the Brassicaceae family, and their suitable use in different biotechnological applications for agriculture [40,41], or those related to the cosmeceutical [36,37] or nutraceutical [42] industries. These vesicles are thermodynamically stable [43] and, among other reasons, this stability was associated with aquaporins [31], which are intrinsic membrane proteins that are also related to the ability of membrane vesicles to stabilize compounds, such as the glucosinolate GRA [33]. Furthermore, membrane vesicles from plants were described to interact with human membrane cells and to cross the stratum corneum, the most superficial skin layer [37]. In addition, two recent works showed that they were used to encapsulate a pomegranate extract to enhance their anti-oxidant effect when applied to human keratinocytes under UV-radiation [36], and to encapsulate SFN in broccoli membrane vesicles (BM-vesicles), improving its anti-proliferative activity [39]. Lastly, the vesicle-specific activity due to GLS, ITCs or certain proteins present in the BM-vesicles after the isolation procedure was demonstrated by Yepes-Molina et al. [39]. Thus, membrane vesicles isolated from *Brassicas* can not only act as carriers but also have activity by themselves 

The aim of this study was to characterize SFN-loaded broccoli membrane vesicles and analyze their potential clinical applications, such as the modulation of inflammatory responses in humans. For this, the system was physicochemically analyzed, the stabilization of SFN in BM-vesicles was studied through the interaction with aquaporins of membrane vesicles and the kinetics of drug release in vitro was determined. Moreover, the ability of the system to modulate the inflammatory response was tested in vitro in a human macrophage model (HL-60 cells), as during inflammation, macrophages play a vital role in immunomodulation through the regulation of the production of several pro-inflammatory cytokines (TNF-α, IL-1β and IL-6).

## 2. Results

### 2.1. Formulation Development

**Figure 1:**
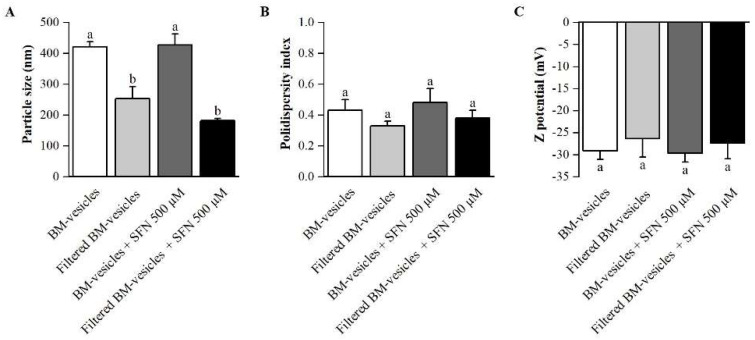
Physicochemical characterization of broccoli membrane (BM)-vesicles. Particle size (**A**), polydispersity index (**B**) and zeta potential (**C**) of the BM-vesicles, BM-vesicles after filtration through a 0.22 µm filter and both loaded with sulforaphane (SFN) were measured. Columns with different letters indicate significant differences between samples according to a one-way ANOVA followed by a post hoc Tukey’s HSD test (*p*-value < 0.05). Values are represented as mean ± SE (*n* = 3).

BM-vesicles were isolated from broccoli leaves to create the nanocarriers. BM-vesicles were physicochemically characterized before and after filtering, which is necessary, on the one hand, to achieve optimal sterility for their subsequent application in cell cultures and, on the other hand, to obtain a population of vesicles that were homogenous in size. The mean particle size, polydispersity index and zeta potential are crucial parameters for the development of a drug delivery system. The results obtained from the Dynamic Light Scattering (DLS) measurements (Figure 1) revealed that the BM-vesicles had a mean particle size of 420 nm with a polydispersity index (PdI) of 0.4 and a zeta potential of around −29 mV. After filtering, the size of the BM-vesicles was significantly reduced to 250 nm, although the PdI and zeta potential did not show statistically significant differences. Once the nanocarriers were characterized, the same measurements were performed after their loading with SFN with respect to the filtration. In this sense, no significant differences were found in any parameters of the BM-vesicles when SFN was encapsulated in comparison with BM-vesicles without SFN (Figure 1).

**Figure 2:**
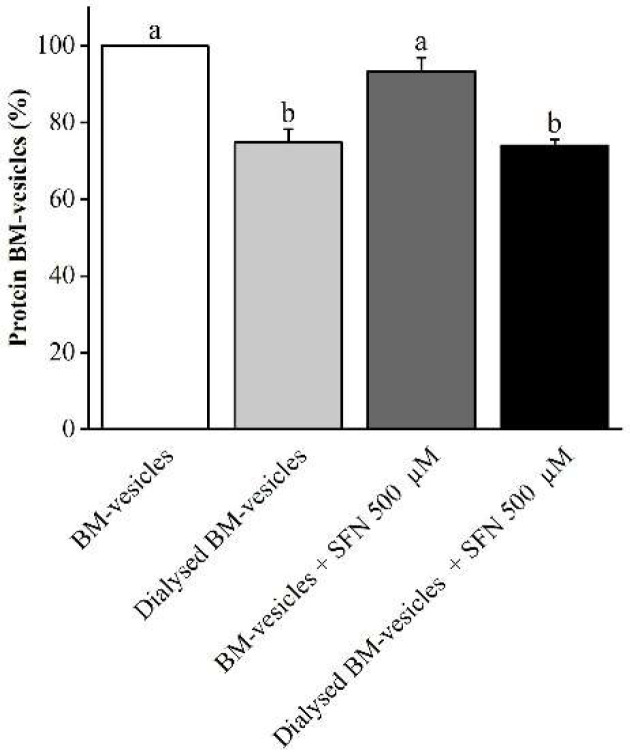
Effect of dialysis and encapsulation on the protein content of broccoli membrane (BM)-vesicles. The protein content in the BM-vesicles was measured after dialysis and SFN encapsulation and referred to BM-vesicles before dialysis and without SFN (valued as 100%). Columns with different letters indicate significant differences between samples according to a one-way ANOVA followed by Tukey’s HSD post hoc test (*p*-value < 0.05). Values are represented as mean ± SE (*n* = 3).

The protein content in the BM-vesicles was checked at every step in the formulation process. The protein concentration in the BM-vesicles was significantly reduced after dialysis (20%) but did not change when the SFN was encapsulated by them (Figure 2).

### 2.2. Drug Encapsulation Studies

**Figure 3:**
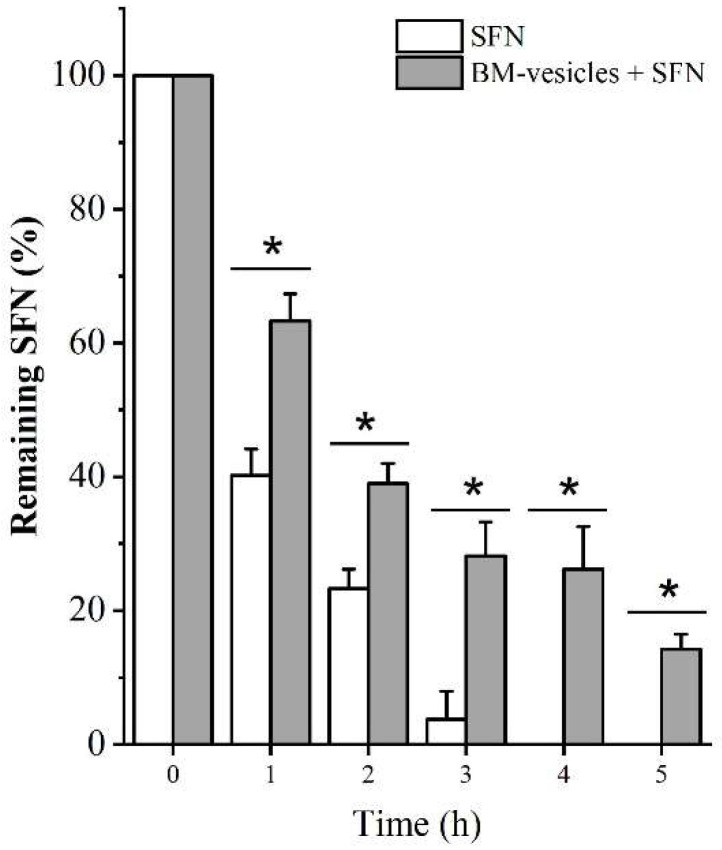
Entrapment efficiency of sulforaphane (SFN) after dialysis. The remaining SFN levels in the dialysis bag were measured in free (SFN, empty bars) and encapsulated form (broccoli membrane (BM)-vesicles + SFN, grey bars) through the dialysis process at different times. Asterisks indicate significant differences between SFN and BM-vesicles + SFN at each time point according to Student’s *t*-test (*p*-value < 0.05). Values are represented as mean ± SD (*n* = 3).

SFN encapsulation was quantified using cycloreactions between ITC and 1,2-benezenedithiol (BDT) [44]. The prepared SFN-loaded BM-vesicles and free SFN were dialyzed at a 1:100 sample/sink ratio and samples were taken every hour to determine the entrapment efficiency of SFN into BM-vesicles. Figure 3 shows the remaining SFN in the dialysis bag at each time point. After 3 h of dialysis, all the free SFN had been released outside of the dialysis bag; meanwhile, when SFN was encapsulated, 3 h after dialysis, the SFN remained in the dialysis bag and, therefore, inside the BM-vesicles. Thus, these data revealed an entrapment efficiency of SFN into BM-vesicles of 28.16 ± 5.05%.

After 2 h of dialysis, the encapsulated SFN started to diffuse from the vesicles, which also acted as a “dialysis membrane”, seeking the osmotic balance between the inside and outside of the vesicle. For this reason, removing non-encapsulated SFN for 1 h will be done in future experiments to prevent the loss of encapsulated SFN from the vesicles during the dialysis process.

Thus, in the formulated system (BM-vesicles with SFN), approximately 35% of the SFN was found outside (non-encapsulated), although it acted quickly and immediately, allowing the encapsulated SFN to remain within the vesicles and be released in a controlled manner.

### 2.3. SFN Quantification in Unloaded and SFN-Loaded BM-Vesicles

The formulated systems (BM-vesicles with and without SFN) were analyzed following the same BDT method to determine the concentration of total ITCs after 1 h of dialysis at a 1:100 sample/sink ratio (Table 1). BM-vesicles by themselves contain ITCs (0.67 µmol mg protein^−1^), and this amount did not change significantly after the dialysis. ITCs were also quantified in BM-vesicles when SFN was encapsulated, and a decrease was observed after dialysis according to the data shown in Figure 3. Thus, the data summarized in Table 1 were used to calculate and adjust the concentration of SFN to the formulations that were assayed in cell cultures.

### 2.4. SFN Interaction with SoPIP2;1 Aquaporin

**Figure 4:**
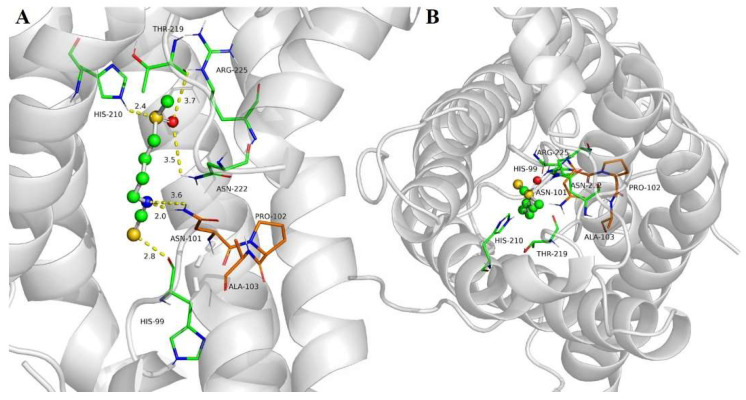
Docking of sulforaphane (SFN) to aquaporin SoPIP2. The aquaporin structural model is shown as grey ribbons, SFN as ball and sticks and selected amino acids of the aquaporin are shown as sticks. The balls are colored depending on the atom type: C, grey; H; white; O, red; N, blue; S, yellow. Side view of the main interactions (**A**) and top view of a protomer from the cytoplasm along the normal membrane (**B**).

The docking of aquaporin SoPIP2;1 with SFN revealed good interactions with the target protein, with a lower coupling free energy of −4.89 kcal/mol, corresponding to an equilibrium dissociation constant (Kd) of 250 µM. SFN bound to aquaporin through electrostatic interactions with HIS-99 and possible hydrogen bonding with ASN-101, HIS-210, ASN-222 and ARG-225 (Figure 4A). Apart from this, non-polar interactions could also play a role in the affinity of SFN with aquaporin, as with THR-219, GLY-220 and ILE-221. Docking calculations suggested that SFN could be located in the water channel pore, interacting with the asparagine–proline–alanine (NPA) motif through ASN-101 (Figure 4B, Video S1).

### 2.5. In Vitro Release Studies

**Figure 5:**
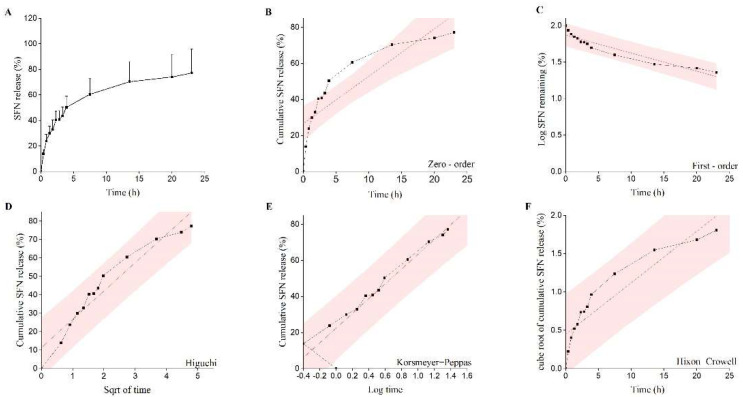
Evaluation of the sulforaphane (SFN) in vitro release profile from encapsulated broccoli membrane (BM)-vesicles. The SFN present in the media after dialysis was evaluated through the dialysis process at different times and referred to the total amount loaded in the BM-vesicles (**A**). SFN release data fitted to various kinetic models: zero-order (**B**), first-order (**C**), Higuchi model (**D**), Korsmeyer–Peppas model (**E**) and Hixon–Crowell model (**F**).

The evaluation of the in vitro drug release from encapsulated BM-vesicles was performed with a dialysis method. The in vitro release behavior of the SFN-loaded BM-vesicles up to 24 h was summarized using the percentage of release, as shown in Figure 5A. SFN was released from the BM-vesicles faster during the first 5 h, after which, it became slower until reaching 80% of the released SFN.

The kinetics and drug release mechanism of the encapsulations was predicted by fitting the release data to several mathematical models of kinetic release (Figure 5B–F and Table 2). The best fit with the highest regression coefficient (R^2^) was found for the equation of the Korsmeyer–Peppas model, with an R^2^ of 0.976. The value of “η” in the Korsmeyer equation predicted that the release mechanism of the drug was 0.33 and, therefore, the release mechanism corresponded to the Fickian diffusion mechanism (Table 2).

### 2.6. Effect of BM-Vesicles and SFN on Cell Viability

**Figure 6:**
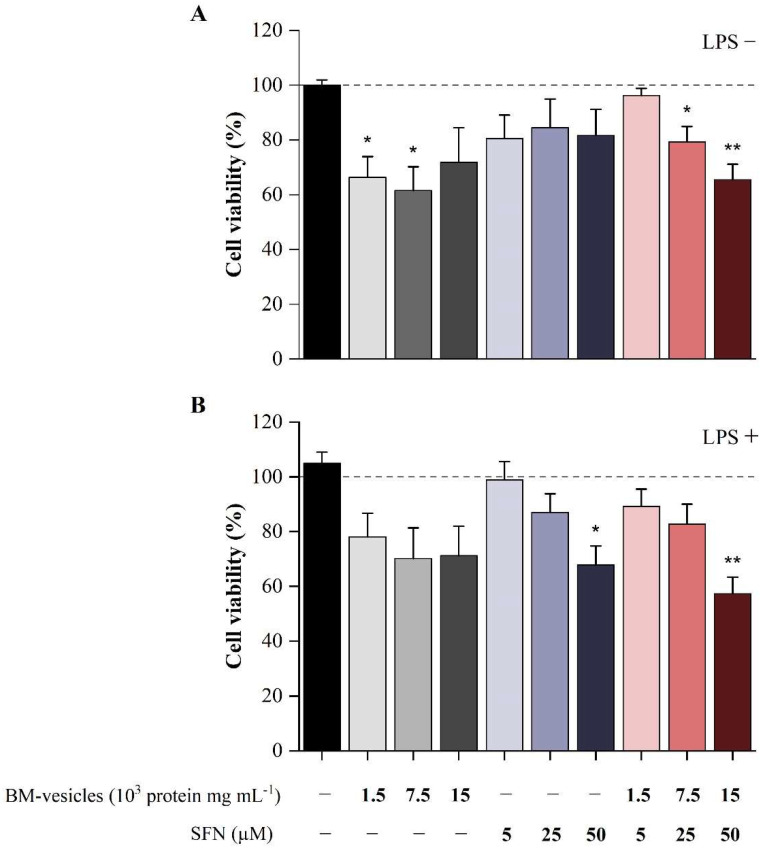
Cell viability of macrophage-like differentiated HL-60 cells exposed to *Brassica*-derived compounds. The cells were treated with broccoli membrane (BM)-vesicles, free sulforaphane (SFN) and SFN-loaded BM-vesicles at different concentrations in basal conditions (**A**) and under inflammatory conditions mediated by lipopolysaccharide (LPS) stimulation (**B**). Data were normalized relative to the unstimulated and untreated control (valued as 100%). Asterisks indicate significant differences between treatments with respect to their reference control (-LPS or +LPS) according to Student’s *t*-test (* *p* < 0.05, ** *p* < 0.01). Values are mean ± SE (*n* = 6).

The effects of different doses of SFN-loaded BM-vesicles, free SFN and unloaded BM-vesicles on cell viability were evaluated in macrophage-like differentiated HL-60 human cells using the MTT assay. This assay was carried out with the macrophage-like differentiated HL-60 cell line, as described in the Materials and Methods section, in basal conditions and under inflammatory conditions induced by the LPS treatment. None of the SFN doses tested showed a significant effect on HL-60 cell viability in basal conditions, while the highest doses of BM-vesicles, either unloaded or SFN-loaded, induced a small but significant reduction of about 30% (Figure 6A). The effect of *Brassica* compounds on cell viability differed slightly under inflammatory conditions (Figure 6B). In this case, only the highest SFN dose (50 µM) of either unloaded or loaded in BM-vesicles affected the cell viability in inflammatory conditions (30% and 40%, respectively). A visual inspection of the cell cultures revealed that the cell morphology and the adhesion capability did not vary after the treatments (data not shown).

### 2.7. Analysis of Anti-Inflammatory Potential of Unloaded BM-Vesicles, SFN and SFN-Loaded BM-Vesicles

**Figure 7:**
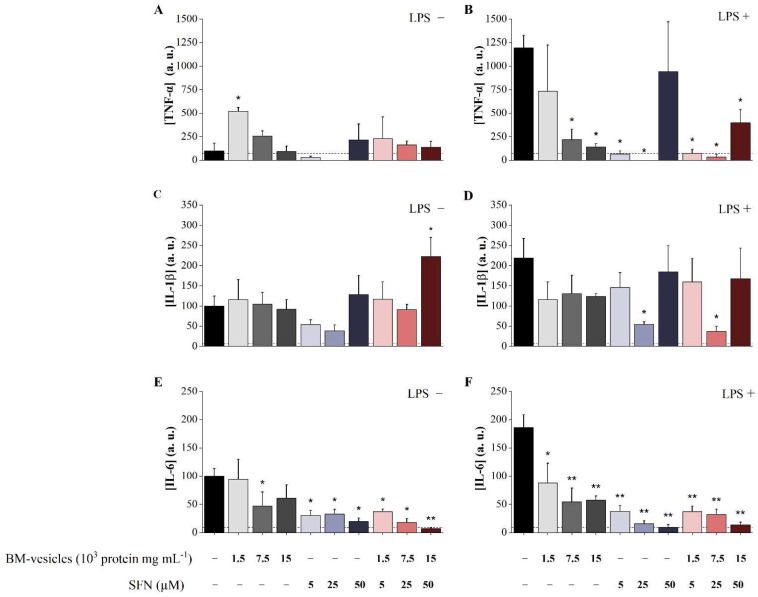
Analysis of the anti-inflammatory potential of sulforaphane (SFN)-loaded broccoli membrane (BM)-vesicles in the HL-60 macrophage cell model. The levels of TNF-α (**A**,**B**), IL-1β (**C**,**D**) and IL-6 (**E**,**F**) secreted by HL-60 cells in response to unloaded BM-vesicles, free SFN and SFN-loaded BM-vesicles were measured in the basal state (**A**,**C**,**E**) and under pro-inflammatory conditions induced by the LPS treatment (**B**,**D**,**F**). Data were normalized relative to the control at the basal level (valued as 100%). Asterisks indicate significant differences between treatments and their reference control according to Student’s *t*-test (* *p* < 0.05, ** *p* < 0.01). Values represent mean ± SE (*n* = 3).

The effects of free SFN, unloaded BM-vesicles and SFN-loaded BM-vesicles on the inflammatory cytokines TNF-α, IL-1β and IL-6 produced by differentiated HL-60 macrophage-like cells were measured in a basal state and under an inflammatory scenario induced by stimulation with LPS (Figure 7).

As shown in Figure 7A, none of the *Brassica* compounds assayed demonstrated significant changes in the level of TNF-α secreted by HL-60 cells in the basal state, with the exception of the increase registered with unloaded BM-vesicles (0.5 µg mL^−1^).

As expected, the LPS treatment of macrophage-like HL-60 cells significantly modified the TNF-α levels, increasing its concentration 11.94-fold in the cell culture (Figure 7B). Under this inflammatory environment, the effect of the compounds on HL-60 cells was evident. In this scenario, free SFN (5 and 25 µM) completely blocked the induction of TNF-α secretion mediated by LPS, while the highest dose of 50 µM did not alter the levels of TNF-α. Unloaded BM-vesicles induced dose-dependent reductions in TNF-α levels, which were statistically significant for the highest concentrations (2.5 and 5 µg mL^−1^). Finally, SFN-loaded BM-vesicles caused a significant reduction in the secretion of this cytokine to the culture medium at all concentrations tested.

Similar to the results obtained for TNF-α, the *Brassica* compounds assayed in macrophage-like HL-60 cells in the basal state did not affect the levels of secreted IL-1β (Figure 7C), with the only exception found in the increase recorded with the highest concentration of the SFN-loaded BM-vesicles (50 µM). Again, free SFN (5 and 25 µM) elicited a reduction in the IL-1β basal levels, although this was not statistically significant.

LPS significantly increased the IL-1β levels (Figure 7D), although to a lesser extent than TNF-α (2.19-fold increase). Under this inflammatory scenario, only 25 µM of SFN, both free and encapsulated in BM-vesicles, drastically reduced the levels of secreted IL-1β, blocking the effect elicited by LPS and reaching even lower concentrations than those of the control at the basal level.

Finally, the effects of *Brassica* compounds on the IL-6 levels secreted to the medium by macrophage-like HL-60 cells were the highest from the three cytokines analyzed. LPS significantly increased the IL-6 levels (Figure 7F) to a similar extent as that found for IL-1β (1.86-fold increase). All the compounds (free SFN, unloaded BM-vesicles and encapsulated SFN) mediated a dose-dependent reduction in IL-6, both in basal conditions (Figure 7E) and the simulated inflammatory condition (Figure 7F), with the effect of encapsulated SFN having the greatest power.

## 3. Discussion

Chronic inflammatory processes have been emphasized because they are closely involved in many diseases, both with a clear inflammatory nature, such as rheumatoid arthritis or cirrhosis, and others, such as diabetes or cancer [2]. In this sense, there is a need to find new therapies to treat these pathologies while avoiding severe adverse side effects. Based on this, the study of bioactive compounds in plants, specifically from the *Brassicaceae* family, is a good starting point and has been exploited in recent years. *Brassica* plants, such as broccoli, are enriched in glucosinolates and isothiocyanates, such as sulforaphane (SFN), which have been widely studied for their anti-inflammatory and anti-cancer roles, among other beneficial aspects for human health [10]. Although SFN is a compound with potential clinical use, it is very unstable, hindering its functionality in real-world applications [23]. In recent years, the encapsulation of SFN into different types of nanocarriers has shown positive results regarding stability in vitro or in vivo [26,28]. In previous studies from our group, membrane vesicles isolated from *Brassicas* were proposed as suitable nanocarriers for use in biotechnological applications [31,37].

Herein, we studied the use of broccoli membrane vesicles (BM-vesicles) with encapsulated SFN as a modulator of the inflammatory response in a human macrophage model (HL-60 cell line). In a first approach, deep characterization of the encapsulation system is needed. The parameters obtained from the dynamic light scattering (DLS) measurement, such as mean particle size, polydispersity index (PdI) or zeta potential, are essential for the nanoencapsulation system development to be used as a new pharmaceutical formulation. These parameters are important, on the one hand, to improve the biodistribution and prolong the pharmacokinetics of encapsulated hydrophobic drugs, such as SFN, and on the other hand, to establish the route of administration of the possible new drug. The BM-vesicles had a size of around 400 nm, which could be suitable for different applications, as particle sizes between 10 and 3000 nm can be used in different applications [45]. Although a smaller size is more versatile and offers more possibilities, and since it is necessary to filter the vesicles to sterilize samples for cell culture applications, this filtration is used to reduce the size of the vesicles. The particle size of the BM-vesicles after filtration was around 200 nm, which is appropriate for a transdermal or intravenous/intramuscular administration, for example, according to the summarized results by Danaei et al. [45]. Furthermore, a low particle size results in a longer blood circulation time when vesicles were administrated intravenously [46]. Regarding the surface charge of BM-vesicles, values close to −30 mV obtained in the DLS measurements suggested that these vesicles were stable [47]. The zeta potential provides valuable information on the physical properties of suspensions, including their stability, and this parameter has been used to optimize encapsulation drug formulations. In this sense, a negative charge has been established as an advantage for topical applications [48,49], which, apart from intramuscular/intravenous administration, could be a suitable way to apply BM-vesicles. Previously, we reported that membrane vesicles cross the stratum corneum [37] and, together with the results obtained regarding the size, charge and PdI, this indicates that BM-vesicles are adequate for use in a topical application.

Size, charge and PdI were also checked when the bioactive *Brassica* compound, namely, SFN, was encapsulated in the vesicles, and no significant changes were detected. Then, assays to determine the percentage of encapsulation of the drug in the vesicles were carried out, with the results showing that 28% of the SFN was strongly encapsulated inside the vesicles, as 20% SFN remained in the vesicles 5 h after dialysis with a high sample/sink ratio (1:100). The determination of the accurate encapsulation percentage in this type of system, which is highly dependent on osmotic equilibrium, and with a dialysis-based method, is very complicated. There are some disadvantages regarding dialysis-based methods, such as the drug released from the vesicles needs to cross an additional barrier before quantification [50,51]; nevertheless, this method provides a correlation with the in vivo release [52] and is cost-effective and simple [50]. Thus, based on the data obtained from fast (1:100 sample/sink ratio) and slow (1:10 sample/sink ratio) release assays, a system with free SFN and a strongly encapsulated SFN after the first dialysis for 1 h was developed for further studies of their effect on human cells.

Likewise, in vitro drug release studies are a crucial step during drug development. These studies are important, as they reflect an approximation of the in vivo performance. Different drug release kinetics have been established since 1987 due to the need for fitting drug release data to mathematical models to determine the release kinetics in liposomes [53]. SFN-loaded BM-vesicles were dialyzed (1:10 sample/sink ratio) for 24 h and the data of drug release obtained fitted to different mathematical models, with the Korsmeyer–Peppas model being the best [54]. Although our vesicles were not pure liposomes, this analysis represented the best approximation to compare with the existing bibliography. Several studies have reported the fitting of the Korsmeyer–Peppas model to drug release data of liposomal formulation, specifically when a release study is carried out using dialysis-based methods [50,53,55,56]. Previously, this model had been shown as time-efficient and precise enough to interpret diffusion data from nanocarriers. Moreover, the advantage of this model is that it allows for describing the drug release mechanism based on the transport exponent (η) [50]. Thus, our system corresponded to a Fickian diffusion mechanism, as η was lower than 0.45, in agreement with other published studies using liposomes with different loaded drugs [57,58,59].

Taking into account the objective of better understanding SFN encapsulation, protein–ligand docking was carried out. Based on this molecular docking data, it was possible to hypothesize that SFN specifically binds to aquaporins. Aquaporins are transmembrane proteins that allow for the movement of water through biological membranes and are shaped by four monomers, with one pore each, that together constitute stable tetramers with a central and functional pore. Aquaporin and membrane vesicle stability were previously related [31,33] and, therefore, special attention was given to these proteins in this study. Aquaporin PIP2;1 from *Spinacia oleracea* (SoPIP2;1) was used as the closest model to *Brassica oleracea* among the crystallized plant aquaporins. The specific interaction of SFN with aquaporin SoPIP2;1 occurred in the pore of the monomers, specifically in the NPA motifs (asparagine–proline–alanine, NPA) through ASN-101. Although SFN can bind to the pore of each monomer, the osmotic water permeability (P*f*) of BM-vesicles when SFN is encapsulated does not change [39]. Since there are no experimental studies where a blockage of the passage of water with SFN was observed, we can speculate that this SFN concentration is not enough to block the water passage and that aquaporin functionality is not affected by SFN encapsulation. Furthermore, the passage of water may occur through the central pore formed by the four monomers [60]. On the other hand, in a previous study, a strong binding between the glucosinolate glucoraphanin and SoPIP2;1 aquaporin was reported [33]. It was also suggested that this union could be key to achieving glucoraphanin stabilization. Hence, aquaporins in vesicles are an important aspect to take into account for the optimization of membrane vesicles as transporters and stabilizers of bioactive compounds.

Based on the results from the in vitro drug release and molecular docking assays, SFN release with two populations with different rate constants could be hypothesized. First, SFN could be released faster through membrane lipids, or the release could be slower through membrane proteins where the SFN can be trapped longer. In this sense, molecular docking studies supported this hypothesis, as they indicated that there could be specific unions with aquaporins. However, it also opened the possibility that SFN could bind to other proteins through electrostatic and van der Waals interactions. In fact, through molecular docking, SFN was shown to bind to serum albumins via non-polar amino acids [61]. Although SFN is a lipophilic drug, at low concentrations, SFN can be found in the aqueous phase due to the presence of heteroatoms, such as S, N and O, in the molecule, which grants the molecule a polar character. This is an important feature for understanding SFN to proteins interactions and, especially in our case, to aquaporins. 

Once the SFN-loaded BM-vesicles were physicochemically characterized, their anti-inflammatory potential was evaluated in a human macrophage-like cell model in vitro with PMA differentiated HL-60 cells. The effect of the *Brassica*-encapsulated SFN was studied in the HL-60 cell model under basal conditions and under an inflammatory scenario that mimicked a chronic inflammatory disease environment. This pro-inflammatory environment was induced via stimulation with LPS (an endotoxin released from Gram-negative bacteria) to trigger the release of pro-inflammatory mediators from the macrophages [62].

First of all, the viability of cells was assayed to discard any potential cytotoxicity derived from the *Brassica* compounds. The results from these assays showed no effect on the cell viability in most conditions tested, although a slight effect at the highest doses of encapsulated SFN (50 µM) was found, which induced a reduction in cell viability of 30%, both in basal and inflammatory conditions. The effect of *Brassica* compounds seemed to differ depending on the inflammatory status of the cells, as BM-vesicles (0.5 and 2.5 µg mL^−1^) affected cell viability only in basal conditions, while free SFN (50 µM) only decreased the LPS-treated cell viability. These data must be considered for the formulation of compounds for in vivo applications.

The release of the inflammatory cytokines TNF-α, IL-1β and IL-6 in macrophage-like differentiated HL-60 cells after stimulation with LPS was already reported [63]. This inflammatory state mimics the environment developed in chronic diseases and provides us with a tool for studying the potential anti-inflammatory effects of BM-vesicles and SFN-loaded BM-vesicles in these experimental conditions, aside from the ones in the basal state with homeostatic conditions. In this sense, SFN-loaded BM-vesicles showed anti-inflammatory activity, reducing or even blocking the increase in TNF-α, IL-1β and IL-6 induced via LPS stimulation, with the effect being more potent for IL-6 and TNF-α, whose levels were drastically reduced even under the basal control levels. In basal conditions, the SFN-loaded BM-vesicles were also able to reduce the IL-6 levels secreted by cells in steady-state conditions. Thus, even if nanocarriers, such as liposomes, normally lack bioactivity [64,65], the BM-vesicles showed bioactivity as they faced several bioactive phytochemicals. We previously reported on the anticancer activity of BM-vesicles against a melanoma cell line [39]. In that work and the current one, ITCs were detected in the vesicles, which could be responsible for the bioactivity of unloaded vesicles, taking into account the bioactivity of BM-vesicles by themselves. Furthermore, from a proteomic study, some proteins with antioxidant activity were identified in BM-vesicles, which could provide health benefits that explain the activity of BM-vesicles on their own [39]. A potent effect, although weaker than in the encapsulated form, was also detected in free SFN and unloaded BM-vesicles on IL-6 levels and TNF-α under the inflammatory scenario. This showed that encapsulation in membrane vesicles did not affect the bioactivity of SFN, which is another important aspect to consider in the design of encapsulation of bioactive compounds in different types of nanocarriers. The anti-inflammatory effect of free SFN detected on macrophage-like HL-60 cells was consistent with data published in other cell models, such as murine macrophages, which showed that SFN treatment reduced the mRNA expression of different inflammatory cytokines via the upregulation of Nrf2 mRNA expression [63], blocking the downstream NFκB pathway [66] or negatively regulating the pro-inflammatory effect of LPS through the inhibition of TLR4-derived cell activation [67]. In other studies, the beneficial effect of SFN encapsulation relative to free SFN was shown in which the delivery system was efficient and had the potential to enhance the therapeutic effect of SFN [26]. In our system, the natural source of the vesicle components made them suitable for in vitro cell studies at different times.

These pro-inflammatory cytokines, which are predominantly produced by activated macrophages, play a key role in the development of the inflammatory response and, therefore, at the onset of chronic inflammation. Altogether, an important factor to highlight from this analysis is that SFN encapsulated in BM-vesicles showed a protective anti-inflammatory activity, as shown by the high levels of TNF-α, IL-1β and IL-6 inhibition with very low cytotoxicity, thus providing a natural alternative for treating chronic inflammatory diseases, such as endometriosis [11], among others [28].

Once a positive effect is determined, another aspect that remains to be discussed is the way in which the vesicles with the compound enter the cells, in this case, macrophages. In previous work, we showed that plant membrane vesicles fused with the plasma membrane of human keratinocytes [37] and, therefore, through the fusion of both membranes, the encapsulated compounds could be delivered inside the cell. Nevertheless, in this study, as it is about macrophages, phagocytosis must be taken into consideration as a possible pathway for the vesicles to enter the cell [68].

Finally, another important aspect to discuss is the possible routes of administration of BM-vesicles loaded with SFN. Several routes were tested for the administration of liposomes, although the parenteral route is the prevalent one for clinically approved treatments [69]. Another possible option is the transdermal route [70], as it was also successfully used for the administration of liposomes in clinical settings. In addition, transdermal treatments against inflammation based on encapsulations specifically concerned us in this study. These are needed and were tested in general terms [71] and in several diseases, such as rheumatoid arthritis [72] and psoriasis [73]. In this sense, membrane vesicles from broccoli plants were characterized for their capacity to cross the stratum corneum [37], which is an essential requirement for the transdermal application of a drug. Lastly, oral delivery is usually not an effective route due to product degradation in the gastrointestinal tract, but recently, Garcia-Ibañez et al. [42] showed that bioactive compounds encapsulated in membrane vesicles from cauliflower were able to exert their specific function after gastrointestinal digestion [42]. Hence, the oral route could also be considered to treat certain pathologies.

## 4. Materials and Methods

### 4.1. Development of SFN-Loaded BM-Vesicles

#### 4.1.1. Plant Culture

Seeds of broccoli plants (*Brassica oleracea* L. var. *Italica*) were pre-hydrated with de-ionized water and continuously aerated for 24 h. After this, the seeds were germinated in vermiculite in the dark at 28 °C for 2 days. They were then transferred to a controlled-environment chamber, and after 5 days, they were placed in 15 L containers with Hoagland’s nutrient solution. The chamber was set up with a 16 h light and 8 h dark cycle, with temperatures of 25 and 20 °C and relative humidity of 60 and 80%, respectively. Photosynthetically active radiation (PAR) of 400 mmol m^−2^ s^−1^ was provided by Pacific LED, WT 470C, LED8OS/840 PSD WB L1600 lights (Philips, Jena, Germany). After 4 weeks of growth, the leaves were harvested for the isolation of BM-vesicles.

#### 4.1.2. BM-Vesicles Isolation and SFN Loading

The leaves were cut into small pieces before vacuum filtering with an extraction buffer (0.5 M sucrose, 1 mM DTT, 50 mM HEPES and 1.37 mM ascorbic acid at pH 7.5) (Sigma-Aldrich, Darmstadt, Germany) supplemented with 0.6% PVP. The mixture was homogenized using a blender and filtered through a nylon mesh (pore diameter of 100 µm). The filtrate was centrifuged (10,000× *g*, 30 min, 4 °C). The supernatant was recovered and ultracentrifuged (100,000× *g*, 35 min, 4 °C), and the pellet obtained was suspended in 500 µL of FAB buffer (5 mM potassium phosphate buffer and 0.25 M sucrose, pH 6.5) and stored at −80 °C until their later use. The protein concentration in the isolated microsomal fraction was determined using the Bradford method [74] with bovine serum albumin (BSA) as the standard. To obtain SFN-loaded BM-vesicles, the drug, at a concentration of 500 µM dissolved in FAB buffer, was mixed with BM-vesicles (0.2 protein mg mL^−1^) with vigorous shaking, reaching a total volume of 2 mL.

### 4.2. Physicochemical Characterization

#### 4.2.1. Determination of Entrapment Efficiency of SFN

The prepared encapsulations were dialyzed in pre-cooled water (4 °C) at a 1:100 sample/sink ratio at 300 rpm in a magnetic stirrer using a 12,000–14,000 molecular weight cutoff (MWCO) dialysis membrane kit (Sigma-Aldrich, Darmstadt, Germany), and samples were taken every hour to determine the entrapment efficiency of SFN in the BM-vesicles. The SFN present in the dialysis bag was quantified using the cyclo-condensation reaction between isothiocyanate (ITC) and 1,2-benzenedithiol (BDT) [44] using an SFN standard curve from 15 to 500 µM. For this, 50 µL sample, 50 µL of potassium phosphate buffer (pH 8.5) containing 1% Triton X-100 and 50 µL of 8 mM BDT in methanol were mixed and heated at 65 °C for 1 h. The solution was cooled to room temperature and the absorbance was measured at 365 nm. The entrapment efficiency (EE) percentage was calculated using the following formula:

(1) $$EE\;\left(\%\right)=\frac{amount\;of\;drug\;encapsulated\;after\;dialysis}{total\;amount\;of\;drug\;added\;for\;encapsulation}\times 100.$$

#### 4.2.2. Particle Size, Zeta Potential and Polydispersity Index Analysis of BM-Vesicles and SFN-Loaded BM-Vesicles

Dynamic light scattering (DLS) was used to measure the particle size, zeta potential and polydispersity index (PdI) at a temperature of 20 °C using a ZetaSizer Nano XL (Malvern Instruments, Malvern, UK). Prior to measurement, the samples were diluted 1:10 with FAB to achieve a suitable concentration to avoid inter-vesicle interactions (count rate of 200 kcps) [75].

#### 4.2.3. In Vitro Drug Release Study

A dialysis method was carried out to study the release kinetics of SFN-loaded BM-vesicles. The prepared encapsulations were dialyzed in the same buffer used to resuspend the BM-vesicles (FAB) at a 1:10 sample/sink ratio using a 14,000 MWCO dialysis membrane kit (Sigma-Aldrich, Darmstadt, Germany) and were stirred at 300 rpm at room temperature. An aliquot was taken at a predetermined time interval and replaced with the same volume of FAB. First, the aliquots were taken every 15 min for the first 1 h and then every half hour for the next 5 h, with the last aliquot taken after 24 h. The detection of released SFN was carried out with the BDT method [44], as described above. The release of SFN from the BM-vesicles was compared with the free SFN suspension.

Finally, with the aim of proposing a release mechanism, data obtained from the in vitro drug release study was fitted to the different drug release kinetic models: zero-order, first-order, Higuchi and Hixon–Crowell. The corresponding linear regression coefficients (R^2^) were determined and the model with an R^2^ closer to 1 was selected as the best-fitting model for the drug release. Moreover, the data were fitted to the Korsmeyer–Peppas model (M_t_/M_∞_ = kt^η^), where “η” represents the drug transport mechanism and can be used to evaluate the mechanism of diffusion (0.45 ≤ η—Fickian diffusion, 0.45 < η < 0.89—non-Fickian diffusion, 0.89—case-II transport and η > 0.89—super case-II transport) [76]. SigmaPlot version 14.5 (Systat Software, San Jose, CA, USA) and OriginPro 2021 (OriginLab Corporation, Northampton, MA, USA) were used to fit the data to the different kinetic models.

### 4.3. Sulforaphane–Aquaporin Binding Studies

Molecular docking of SFN was carried out on aquaporin in the form of a dimer of a tetramer of identical subunits. The chemical structure information of SFN was obtained from the PubChem Substance and Compound database [77] through the unique chemical structure identifier CID: 5350. The molecular structure of aquaporin was taken from the Protein Databank (PDB ID: 4JC6) [78], corresponding to plant aquaporin SoPIP2;1 from spinach (*Spinacia oleracea*) at a 2.15 Å resolution. The input protein structure for docking was prepared by adding all the hydrogen atoms and removing non-functional water molecules. Gasteiger atom charges at pH 7 for both ligand and protein, as well as rotatable bonds in the ligand, were assigned by using AutoDockTools4 software [79,80]. The AutoDock 4.2.6 [79] package was employed for docking. The Lamarkian Genetic Algorithm was chosen to search for the best conformers. The number of independent docks was set to 1000, the maximum number of energy evaluations to 25,000,000 and the population size to 150. Grid parameter files were built using AutoGrid 4.2.6 (La Jolla, California, CA, USA) [81]. The grid box was selected to include the full protein structure containing a total of 8 chains. The grid size was set to 250 × 240 × 320 grid points with a spacing of 0.375 Å. Other AutoDock parameters were used with default values. PyMOL 2.3.0 (Schrödinger LLC, New York, NY, USA) [82] was employed to edit and inspect the docked conformations.

### 4.4. Cell Culture Assays

#### 4.4.1. Preparation of the Compounds to Be Tested in Cell Culture

SFN (500 µM) was encapsulated in BM-vesicles (0.2 mg/mL), dialyzed for 1 h, and ITCs and proteins were quantified after dialysis as described above. Three stock solutions corresponding to free SFN, unloaded BM-vesicles and SFN-loaded BM-vesicles were prepared and diluted in complete culture medium (CCM) for human cell culture to reach the following final concentrations in the medium after filtration through a 0.22 µm pore size filter (Millipore, Darmstadt, Germany) and to achieve optimal sterility and a homogeneously sized population of vesicles. Protein was quantified after filtration using the Bradford method [74] with BSA as the standard to establish the following final concentrations:

1. Free SFN: (a) 5 µM, (b) 25 µM and (c) 50 µM.
2. BM-vesicles: (a) 0.0005 protein mg mL^−1^, (b) 0.0025 protein mg mL^−1^ and (c) 0.005 protein mg mL^−1^.
3. SFN-loaded BM-vesicles: (a) SFN 5 µM + BM-vesicles 0.0005 protein mg mL^−1^, (b) SFN 25 µM + BM-vesicles 0.0025 protein mg mL^−1^ and (c) SFN 50 µM + BM-vesicles 0.005 protein mg mL^−1^.

#### 4.4.2. Cell Culture and Differentiation

For the in vitro model, we used the HL-60 (ATCC^®^ CCL-240™, Manassas, VA, USA) cell line from a human’s promyelocytic leukemia [83]. These cells were first cultured in suspension in complete culture medium (CCM) consisting of RPMI 1640 culture medium (Biowest, Nuaillé, France) containing 10% fetal bovine serum (Biowest, Nuaillé, France) and 1% penicillin/streptomycin (GIBCO, Invitrogen, Paisley, UK) into an incubator at 37 °C in 5% CO_2_.

Once the cells were growing at an exponential rate, they were differentiated into macrophage-like cells by culturing them in 96-well plates at 2 × 10^5^ cells/well in 100 μL of CCM in the presence of 0.1 μg mL^−1^ phorbol myristate acetate (PMA) for 24 h. The cells were then reconditioned by changing the previous medium for 100 µL of fresh CCM, and once again incubated under the same conditions for another 24 h.

#### 4.4.3. Cell Stimulation with LPS and Treatment Application

After differentiation and resting periods, cells were first pretreated with the different doses of the compounds (unloaded BM-vesicles, free SFN and SFN-loaded BM-vesicles) for 30 min at 37 °C. Then, bacterial lipopolysaccharide (LPS) (*Escherichia coli* 0111.B4; Sigma Chemical Co. Saint Luis, MO, USA) was added to a final concentration of 0.1 μg mL^−1^ to induce pro-inflammatory conditions similar to those present in chronic inflammatory diseases and incubated for 24 h at 37 °C in 5% CO_2_. Then, the supernatant was removed and stored at −20 °C for the later measurement of cytokines levels and the viability of the cell culture was evaluated.

#### 4.4.4. Cell Viability Assay

Cell viability in the presence of BM-vesicles and SFN, either free or encapsulated in the vesicles, was evaluated with an MTT assay consisting of the determination of the reduction of 3-(4,5-dimethylthiazol-2-yl)-2,5-diphenyltetrazolium bromide, which is a yellow tetrazole, to purple formazan by cellular mitochondrial enzymes [84]. Briefly, after the incubation time with the selected stimuli, MTT (Alfa Aesar, Thermo Fisher, Karlsruhe, Germany) was added to the cells at a final concentration of 0.3 mg mL^−1^ and incubated at 37 °C in 5% CO_2_ for 2 h. Then, the cells were lysed with a lysis solution (an acidified isopropanol solution containing 0.1% NP-40 detergent and hydrochloric acid at 0.04 M) and the insoluble purple formazan product retained in the cells was solubilized, generating a homogeneously colored solution. The absorbance in the wells was measured at 550 nm with a SPECTROstar Nano spectrophotometer (BMG LABTECH, Ortenberg, Baden-Wuerttemberg, Germany). The percentage cell viability was obtained relative to the control conditions (100% viability, 0% cytotoxicity) according to the following equation:

(2) $$Cell\;viability\;\left(\%\right)=\frac{{\left(Abs_{550nm}\right)}_{sample\;}}{{\left(Abs_{550nm}\right)}_{control\;}}\times 100.$$

#### 4.4.5. Cytokine Production Assays

The detection and quantification of cytokines IL-6, IL-1β and TNF-α present in the supernatants were performed using enzyme-linked immunosorbent assay (ELISA) kits according to the manufacturer’s instructions (Invitrogen, Thermo Fisher Scientific, Waltham, MA, USA). The absorbance in the wells (Immuno Clear Standard Modules, Thermo Scientific strip plates, Waltham, MA, USA) was measured at 450 nm, subtracting the values at 570 nm from the SPECTROstar Nano microplate reader (BMG LABTECH, Ortenberg, Baden-Wuerttemberg, Germany). The cytokines concentration in the cell culture supernatants was calculated using the corresponding standard curve. The modulation of cytokine expression in response to the plant compounds assayed was calculated, normalizing the results by referring the results obtained to control values in each experiment, which were valued as 100.

### 4.5. Statistical Analysis

The R software [85] and GraphPad Prism version 9.1.1 (GraphPad, Chicago, IL, USA) were used to analyze the data and present the results. Statistical differences were determined using Student’s *t*-test or one-way ANOVA, followed by Tukey’s HSD test. Differences were considered to be significant when *p* < 0.05. All the experiments were carried out three independent times and the results are presented as the mean ± SE.

## 5. Conclusions

In the present study, we characterized SFN-loaded BM-vesicles to investigate their potential modulation of the inflammatory response in humans. The physicochemical characterization of the system measured, such as PdI, zeta potential and entrapment efficiency, of SFN-loaded BM-vesicles revealed them to have suitable delivery properties. In addition, the in vitro release model of SFN from BM-vesicles proposed and supported by experimental data and the molecular docking study between SFN and an aquaporin revealed a specific interaction through aquaporin NPA motif. Therefore, there were two different phases of release since SFN could be first released faster through membrane lipids and afterward slower due to the interaction with aquaporin membrane proteins. Therefore, the interaction of SFN with aquaporins could be the key to the sustained effectiveness of SFN due to the slow release, together with phagocytic activity. Finally, the anti-inflammatory potential of SFN-loaded BM-vesicles in a human macrophage-like cell model in vitro with PMA differentiated HL-60 cells provided significant inhibition of TNF-α, IL-1β and IL-6 levels with very low cytotoxicity, indicating their potential use for pharmaceutical uses.

## Figures and Tables

**Table 1:** ITC contents (µM and µmol mg protein^−1^ in a total volume of 2 mL) in BM-vesicles and BM-vesicles + SFN 500 µM before and after dialysis for 1 h at a 1:100 sample/sink ratio. Asterisks indicate significant differences and n.s. indicates non-significant differences between pre- and post-dialysis according to Student’s *t*-test (* *p*-value < 0.05). Values represent mean ± SE (*n* = 3).

ITCs (µM)			
	Pre-dialysis	Post-dialysis	
BM-vesicles	98.22 ± 6.79	91.45 ± 10.28	n.s.
BM-vesicles + SFN 500 µM	495.59 ± 10.51	285.81 ± 13.95	*
ITCs (µmol mg protein^−1^)			
	Pre-dialysis	Post-dialysis	
BM-vesicles	0.67 ± 0.04	0.79 ± 0.09	n.s.
BM-vesicles + SFN 500 µM	3.19 ± 0.07	2.46 ± 0.12	*

Abbreviations: BM, broccoli membrane; ITCs, isothiocyanates; SFN, sulforaphane.

**Table 2:** In vitro release modeling for SFN-loaded in BM-vesicles.

Release Model	Equation	R^2^	η
Zero-order model	M_t_/M_∞_ = K_0_t+ C	0.746	
First-order model	ln(1 − M_t_/M_∞_) = K_1_t + C	0.727	-
Higuchi model	M_t_/M_∞_ = K_H_t^1/2^ + C	0.847	-
Hixon−Crowell model	(1 − M_t_/M_∞_)^1/3^ = K_S_t + C	0.601	-
Korsmeyer−Peppas model	M_t_/M_∞_ = K_K_t^η^	0.976	0.33 ± 0.02

Abbreviations: t, time; t^1/2^, square root of time; C, drug concentration at time t; K_0_, K_1_, K_H_, K_S_, K_k_, release rate constants; η, release exponent; M_t_/M_∞_, cumulative release rate.

## Data Availability

Not applicable.

## Supplementary Materials

The following supporting information can be downloaded from https://www.mdpi.com/article/10.3390/ijms23041940/s1.
